# The ratio of *Matriptase*/*HAI-1 *mRNA is higher in colorectal cancer adenomas and carcinomas than corresponding tissue from control individuals

**DOI:** 10.1186/1471-2407-6-176

**Published:** 2006-07-04

**Authors:** Lotte K Vogel, Mona Sæbø, Camilla F Skjelbred, Kathrine Abell, Esben DK Pedersen, Ulla Vogel, Elin H Kure

**Affiliations:** 1 Department of Medical Biochemistry and Genetics, University of Copenhagen, Blegdamsvej 3, Denmark; 2 Telemark University College, Faculty of Arts and Sciences, Bø i Telemark, Norway; 3 Department of Laboratory Medicine, Section of Medical Genetics, Telemark Hospital, Skien, Norway; 4 National Institute of Occupational Health, Copenhagen, Denmark; 5 Department of Pathology, Ullevaal University Hospital, Oslo, Norway

## Abstract

**Background:**

It has recently been shown that overexpression of the serine protease, matriptase, in transgenic mice causes a dramatically increased frequency of carcinoma formation. Overexpression of HAI-1 and matriptase together changed the frequency of carcinoma formation to normal. This suggests that the ratio of matriptase to HAI-1 influences the malignant progression. The aim of this study has been to determine the ratio of *matriptase *to *HAI-1 *mRNA expression in affected and normal tissue from individuals with colorectal cancer adenomas and carcinomas as well as in healthy individuals, in order to determine at which stages a dysregulated ratio of *matriptase*/*HAI-1 *mRNA is present during carcinogenesis.

**Methods:**

Using quantitative RT-PCR, we have determined the mRNA levels for *matriptase *and *HAI-1 *in colorectal cancer tissue (n = 9), severe dysplasia (n = 15), mild/moderate dysplasia (n = 21) and in normal tissue from the same individuals. In addition, corresponding tissue was examined from healthy volunteers (n = 10). *Matriptase *and *HAI-1 *mRNA levels were normalized to *β-actin*.

**Results:**

*Matriptase *mRNA level was lower in carcinomas compared to normal tissue from healthy individuals (p < 0.01). In accordance with this, the matriptase mRNA level was also lower in adenomas/carcinomas combined as compared to their adjacent normal tissue (p < 0.01). HAI-1 mRNA levels in both normal and affected tissue from individuals with severe dysplasia or carcinomas and in affected tissue with mild/moderate dysplasia were all significantly lower than mRNA levels observed in corresponding tissue from healthy control individuals. HAI-1 mRNA was lower in carcinomas as compared to normal tissue from healthy individuals (p < 0.001). *HAI-1 *mRNA levels were significantly lower in tissue displaying mild/moderate (p < 0.001) and severe (p < 0.01) dysplasia compared to normal tissue from the same patients. Both adenomas and carcinomas displayed a significantly different *matriptase*/*HAI-1 *mRNA ratio than corresponding normal tissue from healthy control individuals (p < 0.05). In addition statistically significant difference (p < 0.001) could be observed between mild/moderate and severe adenomas and their adjacent normal tissue.

**Conclusion:**

Our results show that dysregulation of the *matriptase*/*HAI-1 *mRNA ratio occurs early during carcinogenesis. Future studies are required to clarify whether the dysregulated *matriptase*/*HAI-1 *ratio was causing the malignant progression or is a consequence of the same.

## Background

Matriptase also known as MT-SP1, epithin, TADG-15 and SNC19, is the prototypic member of a recently identified matriptase subfamily of type II transmembrane serine proteases together with matriptase-2 and matriptase-3 [1-7]. It has a multi-domain structure, including an amino-terminal cytoplasmic tail, a transmembrane region, a sperm protein, enterokinase, and agrin (SEA) domain, two complement subcomponents C1r/Cs, urchin embryonic growth factor and bone morphogenic protein (CUB) domain, four low density lipoprotein receptor class A repeats, and a carboxyl-terminal serine protease domain [1,2,4,8]. Matriptase is required for postnatal survival [9] and is essential for processing of pro-filaggrin which is important for epidermal differentiation [10]. Matriptase is co-expressed with its cognate inhibitor, hepatocyte growth factor activator inhibitor 1 (HAI-1) in many types of normal and malignant tissues of epithelial origin [11-14]. HAI-1 is a type 1 integral membrane, Kunitz-type serine protease inhibitor [15] and contains two Kunitz domains and a low density lipoprotein receptor class A domain. *HAI-1 *knock-out mice die during the embryonic development [16].

It has recently been shown that matriptase possesses a strong oncogenic potential since even a modest overexpression in the skin of transgenic mice caused 100% of the mice to develop tumors, 70% of which progressed into carcinomas [17]. Increased expression of HAI-1, completely negated the oncogenic effects of matriptase overexpression [17]. These data strongly suggest that a dysregulated ratio of matriptase to HAI-1 causes malignant transformation to occur at a high frequency whereas aberrant expression of matriptase or HAI-1 *per se *does not seem to be important.

Several studies suggest that matriptase is over-expressed in a wide variety of malignant tumors including prostate, ovarian, uterine, colon, epithelial-type mesothelioma and cervical cell carcinoma [4,12,18-22]. It has also been reported that HAI-1 is over-expressed in breast cancer [23]. However, none of these studies has focused on the ratio of matriptase/HAI-1 expression.

Prompted by the finding that a dysregulated ratio of matriptase to HAI-1 in mice results in tumor formation and carcinogenesis, we have analysed the ratio of matriptase/HAI-1 mRNA expression during colorectal cancer carcinogenesis in humans.

## Methods

### Subject population

The KAM cohort (Kolorektal cancer, Arv og Miljø) is based on the screening group of the Norwegian Colorectal Cancer Prevention study (The NORCCAP study) in the county of Telemark, Norway [24]. The ID number for the NORCCAP study at Clinicaltrials.gov is [I NCT00119912] [25]. A total of 20,780 men and women, age distribution 50–64 years, drawn randomly from the population registries in Oslo (urban) and the county of Telemark (mixed urban and rural) were invited to have a flexible sigmoidoscopy screening examination with or without (1:1) an additional faecal occult blood test (FOBT). 777 individuals were excluded according to exclusion criteria [26]. The KAM biobank currently consists of 170 colorectal cancer cases, 991 cases with adenomas, and 400 controls. Controls were defined as individuals with normal findings at the flexible sigmoidoscopy screening. The colorectal cancer cases of the KAM biobank consist of patients operated on at Telemark Hospital and Ulleval University Hospital in Oslo. The KAM study is approved by the Regional Ethical Committee and the Norwegian Data Inspectorate. In the present study we have analyzed cases with carcinomas (n = 9), cases with adenomas (n = 36) and controls that were polyp free in the screened region of the colon (n = 10). From individuals with adenomas a sample of control tissue was collected 30 cm from anus. From patients with carcinomas, a sample of control tissue was taken from the surgically removed tissue, as far away from the tumor as possible. Control samples from healthy individuals were taken from individuals where no adenomas or carcinomas could be identified with flexible sigmoidoscopy. The histology of the adenomas was examined independently by two specialist histopathologists in order to determine the tumor stage as either mild/moderate (n = 21) or severe (n = 15). They reached the same conclusion in all cases. The distribution of gender and age among cases with colonic carcinomas and adenomas and controls are shown in Table 1. One case with mild/moderate dysplasia showed no detectable mRNA for either *β-actin*, *matriptase *and *HAI-1 *and was therefore excluded from the study. Individuals with dysplasia, who developed carcinomas and were treated at the same hospital, were registered.

### RT-PCR

Total RNA was purified from tissue as recommended by the manufacturers using Qiagen kit (AH diagnostics, Aarhus, Denmark). The tissue had been stored in liquid N_2 _before RNA purification. RNA purification included a DNAse treatment. The cDNA synthesis was performed on approximately 200 ng RNA per 10 μl using Gold RT-RCR kit (Applied Biosystems, Nærum, Denmark). Quantitative RT-PCR was performed on ABI7500 sequence detection system (Applied Biosystems) in Universal Mastermix (Applied Biosystems) using 160/140 nM probe and 600/300 nM primers for *matriptase *and *HAI-1*, respectively. *Matriptase*: primers were matrip F, 5'GCGCTCCCTGAAGTCCTTT3'; matrip R 5'GTCCTGGGTCCTCTGTACTGTTTT3'; probe, matrip t 5'FAM-TCACCTCAGTGGTGGCTTTCCCCA-BHQ-1-3'. *HAI-1*: primers were HAI 1 1532F 5'CGCGGCATCTCCAAGAAG3'; HAI 1 1651R 5'GAACACTGCGACAGCCATCTC3'; probe, HAI 1 1572T 5'FAM-AAATCCCCATTCCCAGCACAGGCTC-BHQ-1-3'. The *HAI-1 *primers detects both *HAI-1 *and the splice variant *HAI-1B*. Primers were designed using Primer Express v2.0 Software. Primers were designed within different exons and with probes covering the exon-exon border to prevent amplification of genomic DNA. *18S rRNA *and *β-actin *primers and probes were obtained from Applied Biosystems. *Matriptase *and *HAI-1 *primers and probes were obtained from TAC Copenhagen. In a validation experiment using a control sample, a dilution series was produced and assayed for matriptase, HAI-1 and β-actin as described for the comparative C_t _method [27]. When plotted it could be shown that the assays are quantitative over a range of 512-fold dilution for both *matriptase *and *HAI-1 *and that the PCR reactions have similar efficiencies provided that a threshold of 0.2 is used for *matriptase *and *β-actin *while the threshold was 0.025 for *HAI-1*. The threshold is a fixed fluorescence signal level above the baseline. The C_t _value of a sample is determined as the fractional cycle number when the samples fluorescence signal exceeds the threshold. The threshold is thus assay specific, determined in the validation experiment and depends on the background of the individual assay. This implies that even though the amplification curves for *matriptase *and *HAI-1 *were overlapping, indicating that the two genes had the same relative abundance, the normalised *HAI-1 *mRNA level seemed higher due to the lower threshold used.

*Matriptase*, *HAI-1*, *β-actin *and *18S *were quantified separately in triplicates. The average standard deviation on triplicates was 6%. The standard deviation on repeated measurements of the same sample (the control) in separate experiments was 8% and 14% for *matriptase *and *HAI-1 *respectively, indicating the day-to-day variation of the assay. Independent PCR reactions of the same samples yielded a correlation coefficient of 0.88. Negative controls (where the RNA was not converted into cDNA) and positive controls were included in all sets. Essentially the same results were obtained whether the expression was normalised to *β-actin *or *18S *(correlation coefficient of 0.921).

### Statistical analysis

MiniTab Statistical Software, Release 13.1 Xtra (Minitab Inc.) and GraphPad Prism 4 were used for the statistic calculations. The data were not adjusted for sex since the incidence ratio of colorectal cancer between the genders is 1.1 in Norway [28].

## Results

**Figure 1:**
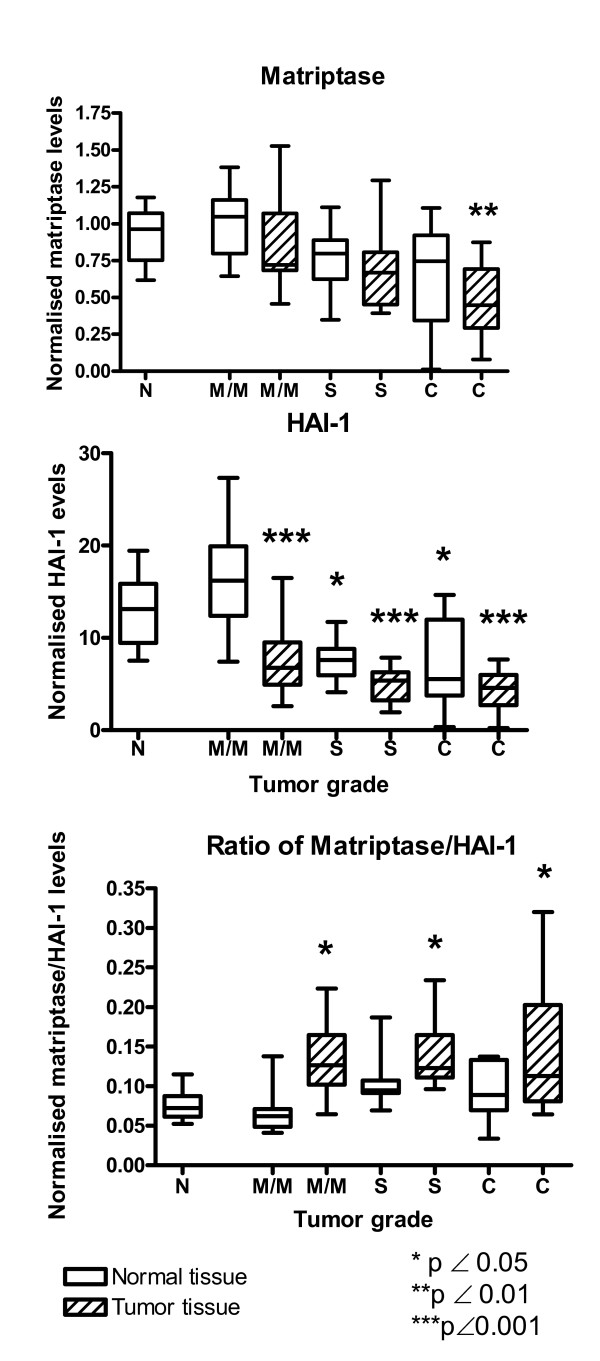
**The mRNA expression of *matriptase*, *HAI-1 *and the ratios between *matriptase*/*HAI-1 *mRNAs determined by real-time RT-PCR from healthy individuals (N), individuals with mild/moderate dysplasia (M/M), severe dysplasia (S) or carcinomas (C). **Both normal (open bars) and affected (hatched bars) tissue were examined from each individual. Both mRNA levels were normalized to the *β-actin *mRNA level. The box shows the interquartile range, the whiskers the range and the median is indicated by a vertical line.

The mRNA levels of *matriptase *and *HAI-1 *were measured in colon tissue samples from healthy control individuals (n = 10) and in healthy and affected tissue from individuals with mild/moderate dysplasia (n = 21), with severe dysplasia (n = 15) and with colorectal cancer (n = 9) by real-time RT-PCR. We chose to normalise the mRNA levels of *matriptase *and *HAI-1 *to the mRNA level of *β-actin*. Essentially identical results were obtained when normalized to *18S *rRNA (see material and methods). We found that *matriptase*, *HAI-1 *and *β-actin *mRNA's are present with almost the same abundances (Fig. 1 and Table 2) within one order of magnitude. This shows that the mRNA's of matriptase and HAI-1 are relative abundant.

The range and the interquartile range of mRNA expression of *matriptase *and *HAI-1 *normalised to *β-actin *are shown in Figure 1. The *matriptase *mRNA level tended to decrease with increasing tumor grade both in dysplastic and cancerous tissue as well as in normal tissue from the same individuals. When the matriptase mRNA level of healthy individuals was compared with colorectal cancer tissue, a statistically significant decrease (p < 0.01) was observed (Table 2 and Fig. 1). Also the *matriptase *mRNA level of all adenomas and carcinomas combined were statistically significantly lower than the corresponding level in healthy tissue from the same individuals (p < 0.01) (Table 2).

*HAI-1 *displayed a stronger general decrease in mRNA level with increasing tumor grade for both dysplastic and cancerous tissues as well as in normal tissue than *matriptase *(Fig. 1). A decrease of 65% was seen in carcinomas as compared to corresponding tissue in healthy control individuals. When comparing the expression level of *HAI-1 *in dysplastic and cancerous tissue with corresponding tissue from healthy individuals, statistically significant lower expression was encountered (p < 0.001) (Table 2) in all groups. There was also a statistically significant difference between normal tissue from healthy individuals and normal tissue from individuals with severe dysplasia and carcinomas (p < 0.05). When comparing affected tissue, with normal tissue from the same individual, we found a statistically significant difference for individuals with mild/moderate dysplasia (p < 0.001) and severe dysplasia (p < 0.01). No statistically significant difference was seen in carcinoma tissue compared to unaffected tissue from the same person (Table 2). A comparison of all adenomas and carcinomas combined, with normal tissues from the same person also showed a statistically significant difference (p < 0.001).

It has recently been shown that is was not the expression of matriptase *per se *but the ratio of matriptase/HAI-1 expression that determines the frequency of carcinoma formation in a mouse model [17]. We therefore investigated the ratio of *matriptase*/*HAI-1 *mRNA expression during colorectal carcinogenesis (Figure 1 and Table 2). We found a statistically significant higher ratio of *matriptase*/*HAI-1 *mRNA levels in tissue with both mild/moderate dysplasia (p < 0.05), severe dysplasia (p < 0.05) and in carcinomas (p < 0.05) when compared to corresponding tissue from healthy individuals. When compared to the ratio of *matriptase*/*HAI-1 *mRNA in normal tissue from the same individual there was a statistically significant difference for mild/moderate dysplasia (p < 0.001), for severe dysplasia (p < 0.001) and for all adenomas and carcinomas combined (p < 0.001). No statistically significant difference was seen in carcinoma tissue compared to unaffected tissue from the same person (Table 2). Thus more unopposed matriptase is present in dysplastic and cancerous tissue than in corresponding tissue from healthy control individuals.

Four individuals with either mild/moderate and/or severe dysplasia later developed colorectal cancer during follow-up. The *matriptase*/*HAI-1 *mRNA levels in the tissue displaying dysplasia of these individuals are shown in Table 3. These individuals did not have higher *matriptase*/*HAI-1 *ratios than the average in their group.

## Discussion

In the present study we have determined the mRNA levels of *matriptase *and *HAI-1 *during colorectal cancer carcinogenesis. It has previously been shown that there is a good correlation between mRNA levels and protein levels for matriptase [12,18,20] and HAI-1 [12].

It has previously been described that matriptase is up-regulated in various human carcinomas by factors of 5–600 [4,12,14,18,19,22,29] and in prostatic tissue with a modest up-regulation [21]. This is in contrast to the present study where we observe a modest but significant 30% down-regulation of *matriptase *mRNA in carcinomas compared to corresponding tissue in healthy individuals. In a very recent study it was reported that *matriptase *mRNA is down-regulated in both gastric and colorectal cancer as compared to adjacent normal tissue [30]. This is in agreement with our observations as the *matriptase *mRNA level tended to decrease with increasing tumor grade.

Since it was recently shown that in mice it is not the matriptase expression *per se*, but the amount of HAI-1 unopposed matriptase that determined the frequency of malignant transformation [17] we also examined the expression of *HAI-1 *mRNA. We found a significant decrease of *HAI-1 *mRNA in tissue with mild/moderate dysplasia and in affected and normal tissue from individuals with severe dysplasia and carcinomas as compared to corresponding tissue from healthy control individuals. A *HAI-1 *down regulation has previously been described in renal cell carcinomas as compared to corresponding normal tissue from control individuals [31]. Recently it was also shown that *HAI-1 *mRNA level is down-regulated in gastric and colorectal cancer as compared to adjacent normal tissue [30]. This agrees well with our findings as also the *HAI-1 *mRNA level tended to decrease with increasing tumor grade.

When examining the ratio of *matriptase *to *HAI-1 *mRNA a significant altered ratio was found in affected tissue displaying mild/moderate dysplasia, severe dysplasia and in carcinomas as compared to corresponding tissue from control individuals. This means that more HAI-1 unopposed matriptase is present in in dysplastic and cancerous tissue, which in a mouse model has been shown to give a increased frequency of malignant transformation [17]. We did not observe any statistically significant differences between normal tissue from healthy individuals and unaffected tissue from individuals with adenomas or carcinomas, although *HAI-1 *mRNA level was statistically significantly lower in normal tissue from individuals with severe dysplasia and carcinomas compared to the level found in healthy persons. A larger number of cases should be studied in order to determine whether the *matriptase*/*HAI-1 *ratio is dysregulated in unaffected tissue from individuals with severe dysplasia and carcinomas. In agreement with our study it was recently reported that the *matriptase */*HAI-1 *mRNA ratio is not significantly different in colorectal cancer compared to adjacent normal tissue [30].

Collectively, this suggests that the dysregulated ratio between matriptase and HAI-1 can arise either by up regulation of the matriptase expression or by down regulation of the HAI-1 expression, or by a combination of the two depending on the type of cancer. Our data further suggest that the disturbance of the matriptase/HAI-1 ratio is an early event in colorectal cancer carcinogenesis as it is already present in tissue displaying mild/moderate dysplasia and that the dysregulation is maintained during all stages of malignant progression. It is interesting to note that even though both *matriptase *and *HAI-1 *is down regulated in colorectal cancer, a similar down regulation takes place in the adjacent normal tissue resulting in an unchanged *matriptase*/*HAI-1 *mRNA ratio.

It is at present unclear how a dysregulated matriptase/HAI-1 ratio is connected to carcinogenesis as the biological function of matriptase is not well understood. It is known that matriptase is essential for processing of pro-filaggrin [10], but this is unlikely to be connected to its role during carcinogenesis. It is also known that matriptase can activate pro-urokinase, protease-activator receptor-2 and hepatocyte growth factor [8,32]. Whether this is important for the ability of unopposed matriptase to cause carcinomas is not clear [17].

The acquisition of the ability to invade surrounding tissue is a key event in carcinogenesis. Proteases are believed to play a crucial role in this process since dissolution of the extracellular matrix is a prerequisite for invasive growth and metastatic spread of tumor cells. Matriptase is possibly involved in this as the serine protease is expressed by the tumor cells themselves and CVS-3983, a matriptase inhibitor, has been developed and shown to retard tumor growth in a human prostate cancer xenograft model [33]. HAI-1 may play an importent role as it has been shown that HAI-1 at the invasive front of colorectal adenocarcinomas is most often in a mature, membrane-bound form, whereas ectodomain shedding is common in the rest of the tumor [34].

Interestingly, it was shown that even a modest dysregulated matriptase expression strongly potentiates chemical carcinogenesis in mice [17]. It was shown that matriptase induced spontaneous carcinoma formation that occurs independently of ras mutations, whereas carcinogen-induced tumors often are accompanied by H-ras or K-ras mutations. The dysregulated matriptase/HAI-1 ratio observed in mild/moderate and severe dysplasia may promote carcinogenesis by potentiating chemical carcinogenesis, and may thus be one of the underlying reasons why polyps transform into carcinomas.

Our results have shown that the abundance of the *matriptase *and *HAI-1 *mRNA are similar to each other and to that of *β-actin *within one order of magnitude. However, it has previously been shown that matriptase protein is present in very low levels in both normal and malignant epithelium (2–24 ng matriptase pr mg detergent-extractable protein) [12]. Likewise HAI-1 is not an abundant protein in mouse intestine (Vogel., L.K unpublished results). This may indicate that these proteins have a short half-life compared to *β-actin *or that the translation frequency of the mRNAs is low. Indeed the half life of HAI-1 when recombinantly expressed in MDCK cells is in the range of a 1.5 hour (Godiksen, S. and Vogel, L.K., unpublished data).

## Conclusion

In conclusion, our study shows that dysregulation of the *matriptase*/*HAI-1 *mRNA ratio as compared to the corresponding tissue in healthy control individuals, is an early event in colorectal cancer carcinogenesis that is maintained during all stages of malignant progression. Future studies are required to clarify whether the dysregulated *matriptase*/*HAI-1 *ratio was causing the malignant progression or is a consequence of the same.

## Competing interests

The author(s) declare that they have no competing interests.

## Authors' contributions

LV and UV conceived the idea of the study. EHK designed the cohort and collected the samples. MS and CFS extracted the RNA, carried out the cDNA synthesis and performed most of the statistical calculations. UV and LV designed and validated primers and probes. LV carried out the RT-PCR and drafted the manuscript. KA and EDKP helped writing the manuscript. All authors helped interpret the results, writing the manuscript and read and approved the final version.

## Pre-publication history

The pre-publication history for this paper can be accessed here:



## Figures and Tables

**Table 1:** Characteristics of cases and healthy persons used in this study

	Healthy	Cases		
		Adenomas^1^		Carcinomas

		Mild/moderate Dysplasia	Severe dysplasia	

	(n = 10)	(n = 20)	(n = 15)	(n = 9)

Men^2^	4	15	13	7
Women^2^	6	5	2	2
Mean age + SD^3^	54.1 ± 3.4	59.1 ± 3.1	58.4 ± 4.3	61.67 ± 9.1

^1^The adenomas are divided in groups according to diagnosed degree of dysplasia (mild/moderate or severe).

^2^There were significantly more males in the group with severe dysplasia compared to the group of healthy individuals (p = 0.014). There was no significant difference in the number of males and females between the group of healthy individuals and the group with mild/medium dysplasia or between the group of healthy individuals and the group with carcinomas (χ^2 ^test).

^3^There are significant differences in age among the four groups of healthy and affected individuals at 95% confidence level. There is no significant difference in age between the three case groups (Mann-Whitney test).

**Table 2:** *Matriptase *and *HAI-1 *mRNA levels in normal and affected tissues. The mRNA levels were normalised to β-actin mRNA levels.

Variable	mRNA level in normal tissue Mean (SD)	P^a^	mRNA level in adenomas/carcinomas Mean (SD)	P^a^	P^b^
**Matriptase**					
Healthy people	0.93 (0.18)				
Mild/moderate	1.02 (0.23)	NS	0.86 (0.31)	NS	NS
Severe	0.74 (0.22)	NS	0.66 (0.24)	NS	NS
Carcinoma	0.66 (0.36)	NS	0.49 (0.25)	<0.01	NS
All adenomas and carcinomas	0.85 (0.29)	ND	0.72 (0.31)	ND	<0.01

**HAI-1**					
Healthy people	12.84 (3.70)				
Mild/moderate	16.35 (5.22)	NS	7.35 (3.67)	<0.001	<0.001
Severe	7.55 (2.12)	<0.05	4.93 (1.83)	<0.001	<0.01
Carcinoma	7.17 (4.79)	<0.05	4.26 (2.25)	<0.001	NS
All adenomas and carcinomas	11.47 (6.23)	ND	5.89 (3.14)	ND	<0.001

**Matriptase/HAI-1 ratio**					
Healthy people	0.076 (0.018)				
Mild/moderate	0.067 (0.024)	NS	0.132 (0.044)	<0.05	<0.001
Severe	0.102 0.027)	NS	0.142 (0.044)	<0.05	<0.001
Carcinoma	0.095 (0.036)	NS	0.144 (0.084)	<0.05	NS
All adenomas and carcinomas	0.085 (0.032)	ND	0.138 (0.053)	ND	<0.001

NS = not significant, ND = not determined

a) p value for the comparison to the expression levels in healthy persons using a one-way ANOVA and Tukey's post test for paired comparisons.

b) p value for the comparison of the expression levels in normal and affected tissue from the same person using a paired T-test

**Table 3:** The *matriptase*/*HAI-1 *ratio of individuals with mild/moderate or severe dysplasia who later developed carcinomas. The mRNA levels were normalised to β-actin mRNA levels.

	*Matriptase*/*HAI-1 *ratio	
KAM ID no.	Mild/moderate	Severe

278	0.124	0.100
344		0.115
803		0.121
193	0.146	
Mean (SD)*	0.132(0.044)	0.142(0.044)

*Mean (SD) of their group also listed in Table 2.
